# Citizen science data reveals the need for keeping garden plant recommendations up-to-date to help pollinators

**DOI:** 10.1038/s41598-020-77537-6

**Published:** 2020-11-24

**Authors:** Helen B. Anderson, Annie Robinson, Advaith Siddharthan, Nirwan Sharma, Helen Bostock, Andrew Salisbury, Stuart Roberts, René van der Wal

**Affiliations:** 1 School of Biological Sciences, University of Aberdeen, Cruickshank Building, Aberdeen, AB24 3UL United Kingdom; 2 Knowledge Media Institute, The Open University, Milton Keynes, UK; 3 RHS Garden Wisley, Woking, GU23 6QB UK; 4 CAER, School of Agriculture, Policy and Development, University of Reading, Reading, UK; 5 Department of Ecology, Swedish University of Agricultural Sciences (SLU), Ulls väg 16, 75651 Uppsala, Sweden

**Keywords:** Ecology, Ecology

## Abstract

Widespread concern over declines in pollinating insects has led to numerous recommendations of which “pollinator-friendly” plants to grow and help turn urban environments into valuable habitat for such important wildlife. Whilst communicated widely by organisations and readily taken up by gardeners, the provenance, accuracy, specificity and timeliness of such recommendations remain unclear. Here we use data (6429 records) gathered through a UK-wide citizen science programme (BeeWatch) to determine food plant use by the nations’ bumblebee species, and show that much of the plant use recorded does not reflect practitioner recommendations: correlation between the practitioners’ bumblebee-friendly plant list (376 plants compiled from 14 different sources) and BeeWatch records (334 plants) was low (r = 0.57), and only marginally higher than the correlation between BeeWatch records and the practitioners’ pollinator-friendly plant list (465 plants from 9 different sources; r = 0.52). We found pollinator-friendly plant lists to lack independence (correlation between practitioners’ bumblebee-friendly and pollinator-friendly lists: r = 0.75), appropriateness and precision, thus failing to recognise the non-binary nature of food-plant preference (bumblebees used many plants, but only in small quantities, e.g. lavender—the most popular plant in the BeeWatch database—constituted, at most, only 11% of records for any one bumblebee species) and stark differences therein among species and pollinator groups. We call for the provision and use of up-to-date dynamic planting recommendations driven by live (citizen science) data, with the possibility to specify pollinator species or group, to powerfully support transformative personal learning journeys and pollinator-friendly management of garden spaces.

## Introduction

The widespread declines in terrestrial insect pollinator numbers^1–3^ and their consequences including the provision of pollination services^4–6^ have attracted much media attention^7,8^. With habitat loss being the main driver of insect declines^9^, pollinator-friendly gardening has been promoted as a societal response to help mitigate and transform urban environments into valuable habitat for such insects^10,11^. A key instrument used is to recommend the planting of specific flowering species thought to be good for pollinators^12,13^. Many resources are available to aid people in doing so, e.g. wildlife gardening books and websites, ready-made seed mixtures for growing wildlife meadows, pollinator-friendly labelling by plant producers and retailers, and lists of plants that are good for pollinators provided by conservation agencies, horticulture societies and garden centres. However, labels that state specific plants are “pollinator-friendly” are generic^14–17^, implying that such plants are similarly beneficial and for all pollinating insects, e.g. bumblebees, honeybees, butterflies, hoverflies and beetles and the different species therein.

Here we show that practitioner lists of pollinator-friendly and bumblebee-friendly plants are remarkably similar, indicating no specificity for individual pollinator group needs. We detail how data contributed—through the citizen science platform BeeWatch—by members of the public across the UK on bumblebee plant use did not match widely available planting recommendations for bumblebees. Furthermore, the citizen science data showed that different bumblebee species favoured different food plants. Whilst using the label ‘pollinator-friendly’ brings to attention the plight of pollinating insects and will sway many to purchase plants that may be used as respective food plants, acknowledging greater complexity, including the existence of different levels of pollinator friendliness and variation among species therein, could create learning journeys and behavioural change^18^. Human–Computer-Interaction approaches can be used to develop interactive ‘planting for pollinators’ tools that support such learning. Such planting recommendations would remain up-to-date if based on species interaction data from active citizen science programmes.

## Results

### Evaluating practitioners’ pollinator- and bumblebee-friendly planting recommendations

**Figure 1:**
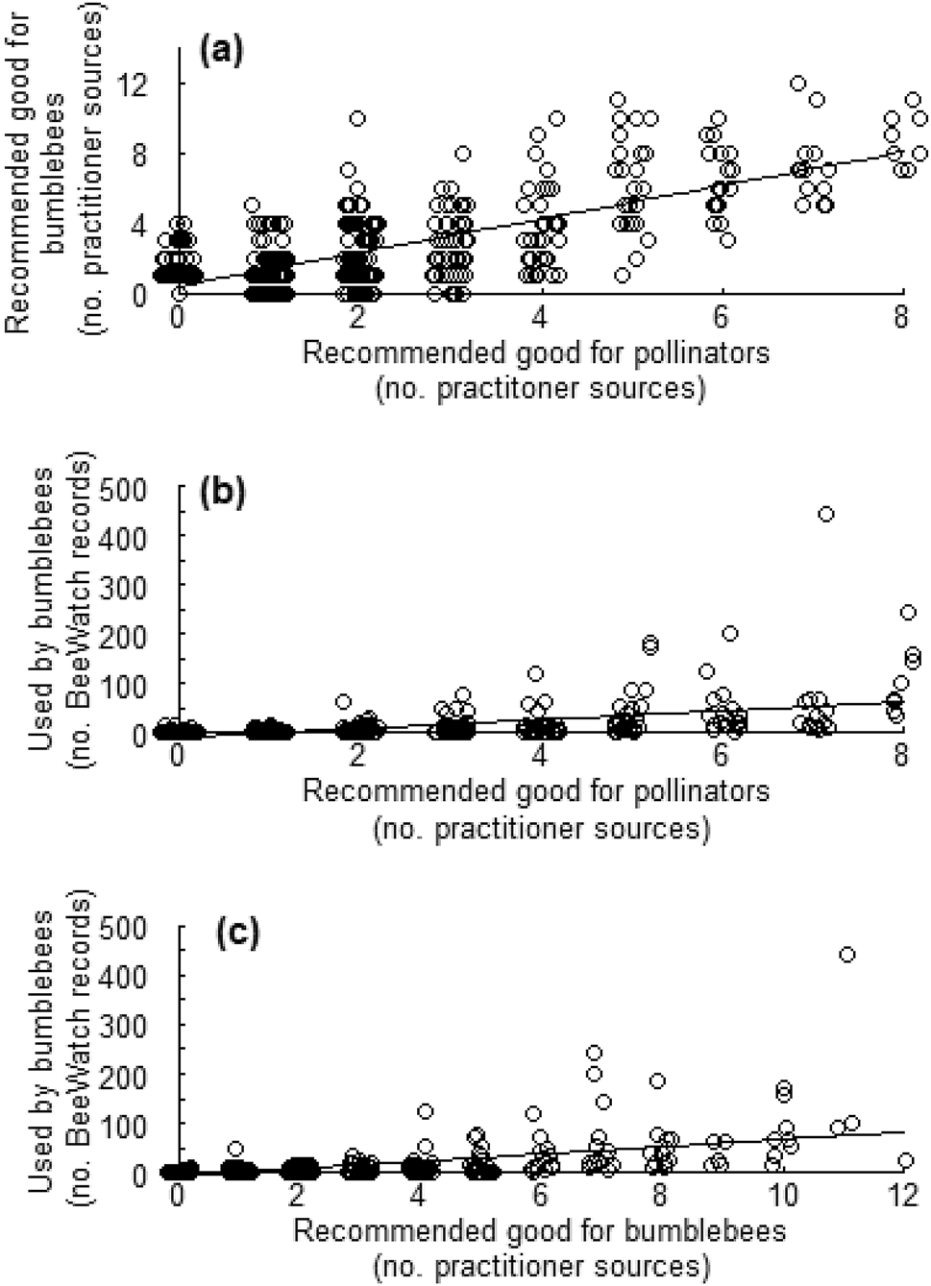
Relationships between the number of different sources of practitioner information recommending certain plant species as being good for pollinators in general and bumblebee specifically, and how frequently such plants have been observed being used by bumblebees based on BeeWatch citizen science data. **(a) **Relationship between the number of practitioner sources that recommended a plant as being good for pollinators in general (total sources = 9; Suppl. Appendix Table 1 sources 1–9) and the number of practitioner sources that recommended a plant as being good for bumblebees specifically (total sources = 14; Suppl. Appendix Table 1, sources 10–23); **(b)** relationship between the number of practitioner sources that recommended a plant as being good for pollinators in general (total sources = 9; Suppl. Appendix Table 1, sources 1–9) and the number of times that plant was recorded as being used by bumblebees by BeeWatch participants, and; **(c) **relationship between the number of practitioner sources that recommended a plant as being good for bumblebees specifically (total sources = 14; Suppl. Appendix Table 1, sources 10–23) and the number of times that plant was recorded as being used by bumblebees by BeeWatch participants. BeeWatch data covers the period August 2011 to June 2017. Due to a large amount of overlap, points in all figures are jittered on the x-axes.

Across all 23 UK practitioner sources consulted (Suppl. Appendix Table 1), 465 different plant species were listed as good for pollinators and 376 plants were recorded as good for bumblebees. Numerous species occurred in both lists, and using those reoccurrences allowed us to reveal a remarkable similarity between those plants regularly mentioned as pollinator-friendly and those repeatedly identified as being bumblebee-friendly plants (r = 0.75, p < 0.001; Fig. 1a). This implies a generality in practitioners’ assumptions about pollinator-friendly plants for bumblebees, i.e. that plants identified as being good for pollinators in general are also considered to be the most suitable for the pollinator subgroup, bumblebees or visa-versa.

**Figure 2:**
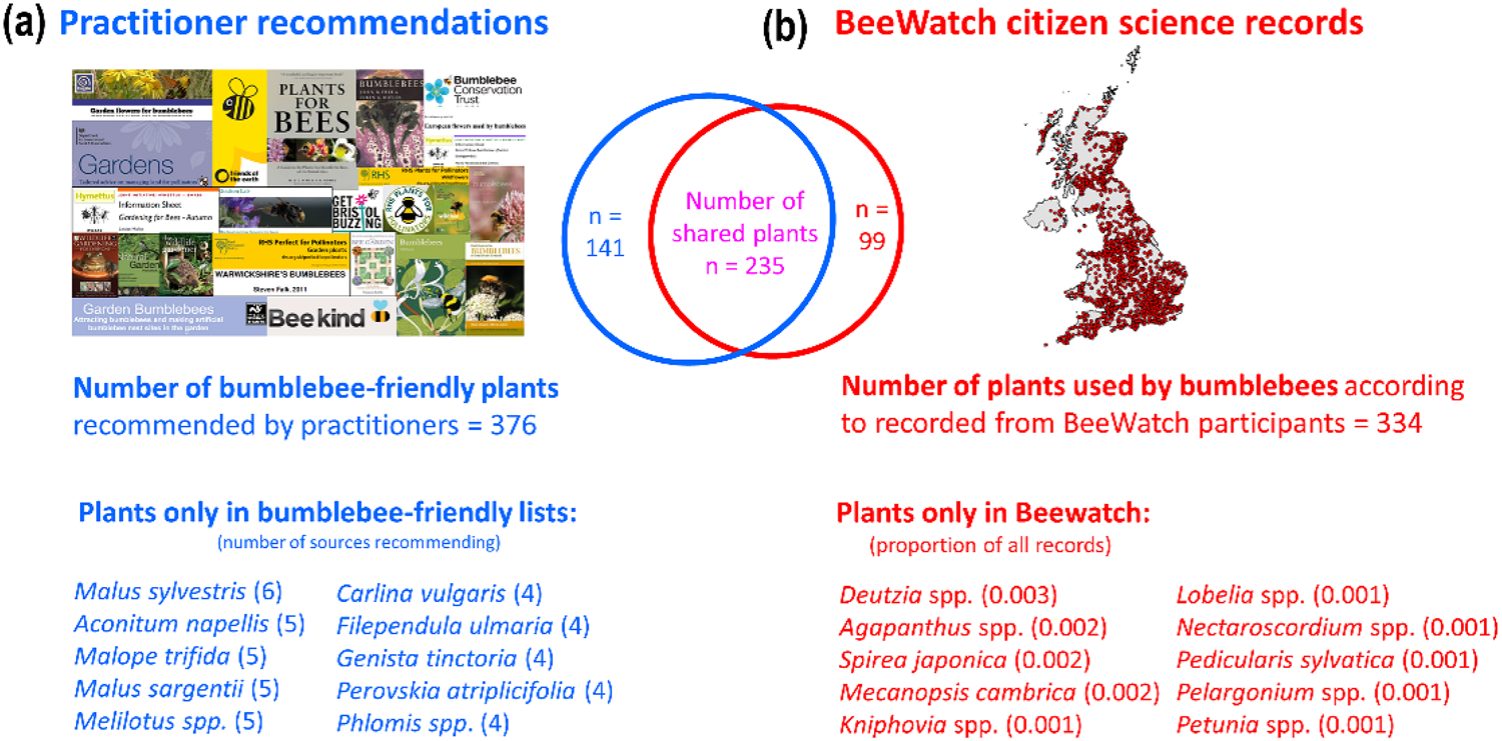
Comparison of data sources: practitioner recommendations versus BeeWatch citizen science data. Images of practitioner sources used for gathering data on pollinator and bumblebee friendly plants and a map of the UK showing all locations for which BeeWatch data were submitted, and schematic showing numbers of plants recommended as bumblebee-friendly by practitioners and number of plants photographed and reported by BeeWatch participants as being used by UK bumblebees (middle). **(a)** Practitioner sources used for gathering data on pollinator- and bumblebee-friendly plants and the total number of plants recommended as bumblebee-friendly (in blue; derived from 14 sources detailed in Suppl. Appendix Table 1 in bold font); **(b)** red symbols show locations of observations of bumblebees feeding on plants across the UK as recorded and photographed by BeeWatch participants between August 2011 and June 2017 and the total number of plants recorded and photographed as being used by UK bumblebees by BeeWatch participants (in red). The number of plants common to both the bumblebee-friendly list and BeeWatch is shown in purple. The top 10 plants recorded in BeeWatch but not found in the bumblebee-friendly list shown in red (number in brackets = their proportions out of the total number of plants recorded). The top 10 plants reported as being good for bumblebees but not recorded by BeeWatch participants shown in blue, number in brackets = number of sources they occurred in, from a total of 14). The UK map contains data from BeeWatch (2011–2017) and OS data Crown copyright and database right (2019) and was generated by H. Anderson using ArcGIS Desktop 10.7 Esri Inc. 1999–2018.

The citizen science programme BeeWatch gathered 6429 records of plant visitation by bumblebees from gardens and public greenspaces across the UK (Fig. 2a). These records associated UK bumblebees with 334 different plant species. Almost three-quarters of those (250 plant species) occurred in the practitioners’ sources identifying species as being good for pollinators in general. Nearly two-thirds of the plants recorded in the BeeWatch database (235) occurred in the practitioners’ list of plants identified as good for bumblebees specifically (Fig. 2b). A high percentage of shared species may suggest a reasonable level of agreement. Yet, when taking into account the frequencies at which plants occurred across all practitioner sources and comparing those recommendations with actual reported levels of plant use by BeeWatch participants, a different picture emerged: relationships were significant but with rather limited predictive power, for both pollinator-friendly (r = 0.52, p < 0.001; Fig. 1b) and bumblebee-friendly plant sets (r = 0.57, p < 0.001; Fig. 1c). Only 12 of the top 25 most frequently practitioner-recommended bumblebee-friendly plants appeared in the top 25 food plants used by bumblebees, as reported by BeeWatch participants (bold font in Suppl. Appendix Table 2). Vice versa, less than half of the top 25 BeeWatch-identified forage plants were in the top 25 bumblebee-friendly plants recommended by practitioners (bold font in Suppl. Appendix Table 2). Thus, there appears to be a discrepancy between plants identified as being important for pollinators in general, or even specifically for bumblebees, by practitioners and those regularly recorded by BeeWatch participants as having been used by bumblebees.

**Table 1:**
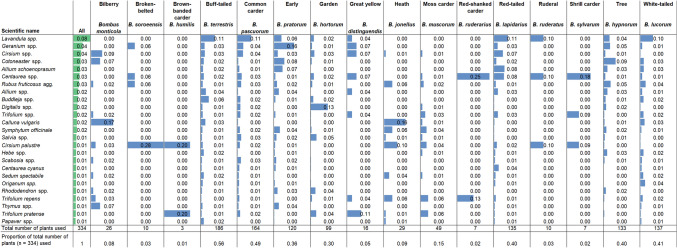
Rank order of the top 25 plants used by bumblebees based on data from the citizen science programme BeeWatch.

For each plant, its relative abundance (i.e. proportion) on the BeeWatch database was calculated, for all bumblebee species combined (‘All’) and for each of the 16 true bumblebee species. Coloured bars are included to aid interpretation, whereby the top plant of a certain bumblebee species attracted the widest colour bar, and the colour bars of the other plants are proportional to that top plant. Where the bar is not the full width of the column, a species not in the overall top 25 list was the most widely used for that bumblebee species. The total number of plants observed (for all bumblebees combined and individual species respectively) is provided in the second row from the bottom. The bottom row details the proportion of plants (from a total of 334) used each individual bumblebee species. Data compiled from information recorded by BeeWatch participants between August 2011 and June 2017 (6429 bumblebee–plant interactions).

Making the assumption that BeeWatch participants had access to the majority of recommended pollinator- and bumblebee-friendly plants, many of these recommended plants were not observed by BeeWatch participants as being exploited by bumblebees (Fig. 2a). An absence, and under-reporting, of specific plant groups was apparent from the BeeWatch data. For instance, more than a quarter (27%) of the 207 plants which either did not appear in the BeeWatch database (e.g. crab apple species (*Malus sylvestris, Malus sargentii*); Fig. 2b) or were infrequently reported (e.g. other trees such as birch (*Salix* spp.), 23 records, and pear *(Pyrus communis*), 2 records) were large shrubs or trees known to exceed 2.5 m in height, where the flowers would be above the line of sight of most people and hence scarcely searched for bumblebees, photographed and submitted to BeeWatch. Many wildflowers were also absent from the BeeWatch database, e.g. monk’s-hood (*Aconitum napellus)* and sweet clovers (*Melilotus* spp.) (Fig. 2b), perhaps because they are poisonous or regarded as ‘weeds’ and therefore not widely grown in gardens (where the majority (80%) of BeeWatch records originated from). Conversely, since there are over 70,000 plants in cultivation in the UK^19^, it is feasible that some species recorded by BeeWatch users as being used by bumblebees were missing from the practitioner-recommendation lists. Some of these absent plants were common garden species, such as lobelia (*Lobelia* spp.), petunia (*Petunia* spp.), red-hot poker (*Kniphovia* spp.), and lily-of-the-Nile (*Agapanthus* spp.) (Fig. 2b), perhaps overlooked by practitioners because of their commonality. Plants that flower outside the normal flight times of bumblebees, e.g. cyclamen (*Cyclamen* spp.) and pasqueflower (*Pulsatilla* spp.) were also reported as having been used by bumblebees by BeeWatch users but were not included on the practitioner-recommended lists. This is presumably an example of these lists being out of sync with the recently developed phenomenon of active over-wintering buff-tailed bumblebees increasingly observed in southern areas of the British Isles^20,21^ as winter temperatures increase.

### Range and diversity of plants used by different bumblebee species

The notion of ‘bumblebee-friendly’ plants implies a high level of commonality among the many different species of bumblebees. Indeed, this appeared to be the case for cuckoo bumblebees—a relatively rare group specialised in parasitizing other bumblebee species—as BeeWatch data for all six cuckoo species were found to rely heavily on a single food plant (marsh thistle (*Cirsium palustre*); Suppl. Appendix Table 3). Yet, only four true bumblebee species [common carder (*Bombus pascuorum*), white-tailed (*B. lucorum* agg.), buff-tailed (*B. terrestris*) and red-tailed bumblebees (*B. lapadarius*)], relied relatively heavily on the most commonly used plant—lavender (*Lavendula* spp.) (Table 1). For the other 12 true bumblebee species, the most frequently used plants were found across the rank order of most commonly used plants (Table 1). Thus, most bumblebee species had distinct food plant preferences, i.e. different species of bumblebee preferred different species of plants. In fact, the BeeWatch data indicates that only the morphologically rather similar white-tailed (*B. lucorum* agg.) and buff-tailed bumblebee (*B. terrestris*) had diets that showed considerable similarity (Table 1).

Although certain plant species were more ‘popular’ than others, the relative proportions accounted for by the top plants for individual bumblebee species in the BeeWatch data were relatively low. For instance, lavender (the main food plant for many bumblebees) and a plant in the top 3 practitioner recommendations (Suppl. Appendix Table 2), only accounted for 11%, at most, of the records for any bumblebee species, and the 7 plants that stood out as being most frequently reported by BeeWatch participants constituted only 3–8% of the total number of plants used by all true bumblebees (Table 1 and Suppl. Appendix Table 2). The bumblebees that had slightly higher percentages of records against their ‘most favourite food plant species’, such as 17% and 16% of records referring to heather (*Calluna vulgaris*) for the billberry bumblebee (*B. monticola*) and heath bumblebee (*B. jonellus*) respectively, were those considered to have a more ‘specialist’ diet^22^. The high number of records for cranesbill (*Geranium* spp.) use by the early bumblebee (*B. pratorum*) and foxglove (*Digitalis* spp.) by the garden bumblebee (*B. hortorum*), 16% and 13% respectively, may be accounted for by their morphology^23^ —open geranium flowers used by the small (and small-tongued) early bumblebee and deep foxgloves used by the long-tongued garden bumblebee.

**Figure 3:**
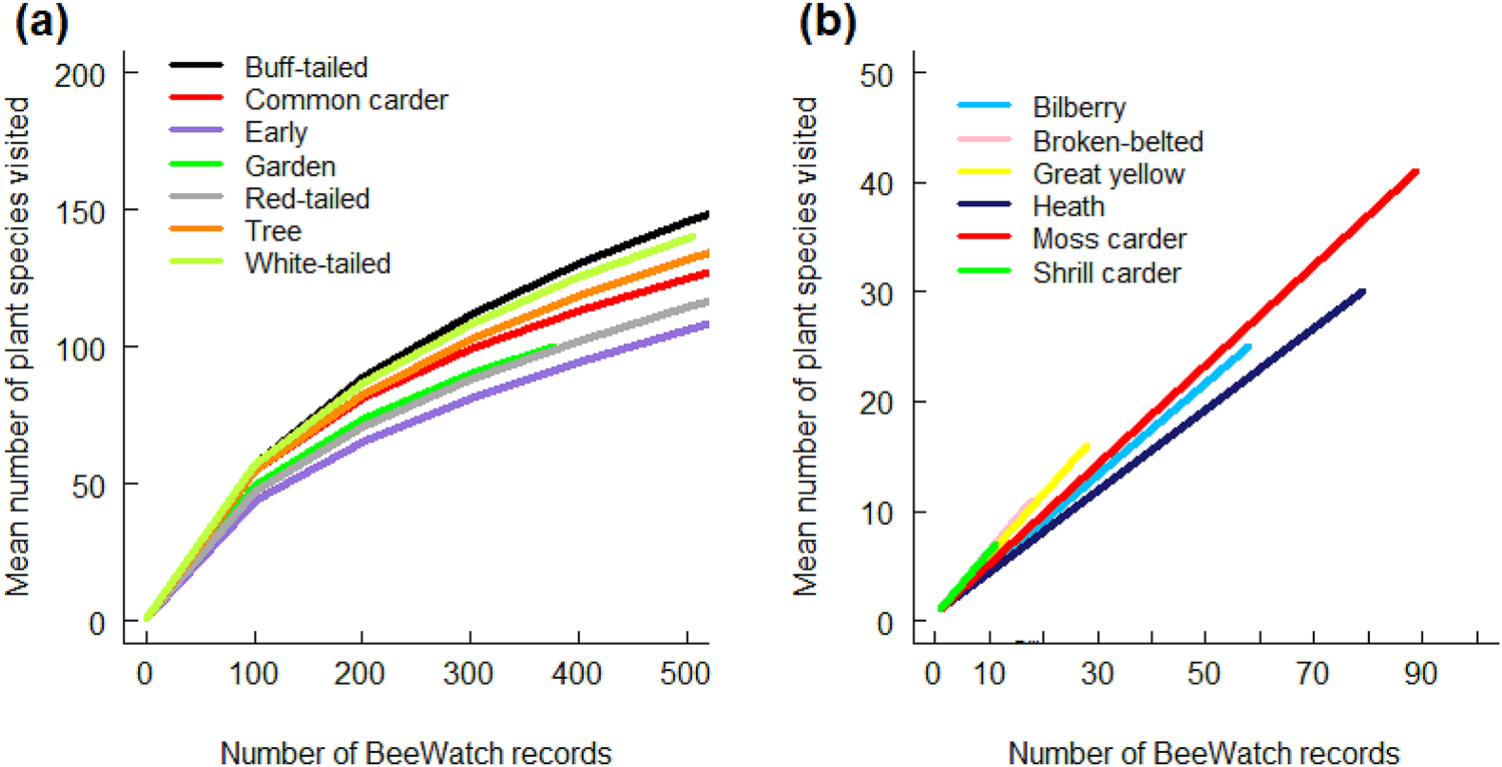
Estimated mean numbers of plants predicted to be visited by bumblebee species when assessing an increasingly large number of records. **(a)** mean number of plant species predicted to be used by each of the seven ‘common’ UK true bumblebee species; **(b)** mean number of plant species predicted to be used by each of six ‘rarer’ UK true bumblebee species. Results were generated using data gathered from the citizen science programme BeeWatch, where 10 plant choices were randomly sub-sampled, without replacement, using 100 repeats of the procedure. Predictions for bumblebee species which had less than 10 records, i.e. the brown-banded carder and the red-shanked carder bees and the ruderal bumblebee, were unable to be made.

Most bumblebee species, however, were found to use a wide range of plants (Table 1). This is in contrast to the recommendations by practitioners, where more than a third of all plant species were recommending by four or more of the 14 sources, showing considerable levels of agreement between different sources as to which plants would be most useful. The use of many different plant species by the most common bumblebee species could be because observations by BeeWatch participants were made over many months: there was a high correlation between the number of months in which different bumblebee species were active and the predicted number of plant species they could potentially use, r = 0.73, p = 0.005. This indicates that these common (and long-flying) bumblebee species had the opportunity to feed on many plants that were in flower at different times during the season. By extension, the rarefaction model predicted that as the sample size of any bumblebee species increased, so did the mean number of plants any one bumblebee species would be expected to use (Fig. 3). This reveals that the label ‘good for bumblebees’ is very likely to be valid for a large number of plant species, however, it may have limited utility as in reality most plant species will be little used (Table 1). In other words, there are degrees of ‘friendliness’, with only a few plant species being used to a greater extent than most others. Furthermore, the set of most-used plants are different for many of the different species of bumblebee.

## Discussion

The need to conserve pollinating insects has captured the public’s attention^24,25^. One method of doing so is through the planting of pollinator-friendly plants. The labelling of such plants is known to work well in certain settings. For instance, in garden centres where people are faced with a huge amount of choice, schemes advocating particular plants as good for pollinators have been very successful^8^. However, most of the “pollinator-friendly” planting recommendations available to the general public are remarkably similar, with the absence of reference to specific evidence detailing when, where and by whom the information was collected, making it difficult to ascertain on what basis such recommendations are made. The omission of reference to underlying data leads to speculation that many of these recommendations lack independence, i.e. that new lists are based on older ones^16^. This may lead to many suggestions being relatively old, with no account taken of changes in popularity of particular garden plants, availability of new plant species, or arrival of new cultivars^17^ with different nectar or pollen qualities (these being the focus of foraging pollinating insects^26^). Mismatches, or more opportune overlaps, of flowering times with insect emergence flight times may now occur due to phenological changes, making planting recommendations out-of-date^27–29^. A further drawback of such schemes lies in the overt communication of a simple dichotomy, i.e. either good for pollinators or not, whilst BeeWatch data—and various other studies—make it clear that there are gradations and pollinator-specific patterns therein^22,23^. Whereas the labelling of plants in a dichotomous manner may be a sufficient entry-point to begin engaging people with pollinators, the self-perpetuation of recommendations on which this dichotomy is based will continue to lead to confusion for individuals who seek out such plants and find that they either do not attract the pollinators they were hoping for or are little used by pollinators. This, in turn, would result in a lack of more in-depth engagement with these societally important insects.

Whilst being more up-to-date, detailed and bumblebee-species specific, data from the citizen science programme BeeWatch is not without problems. Arguably, the most difficult aspect of this citizen science data is that it does not necessarily represent plant attractiveness per se, but instead informs us which plants different bumblebee species used across the UK. Here, the popularity of certain plants, and thus their abundances, will contribute to what species appear attractive (based on their proportion of all BeeWatch data; Fig. 1). Resolving this dilemma at the spatial scales required, along with the number of plants concerned is very difficult, and arguably out of reach for a country-wide citizen science scheme that needs to engage widely with the public. Abundance data of plant species used (and, in the case of BeeWatch, photographed) by bumblebees could be requested from scheme participants, but in the absence of abundance data of alternative forage plants such information is difficult to interpret^30^. More absolute measures of relative attractiveness can be determined through standardised protocols involving timed observations of flowers, ideally in a paired manner within the same locations (i.e. individual gardens). However, this can likely only be done for a limited number of species, and not the many hundreds of recommended (n = 376) or used (n = 334) plants our study deals with. Although realising that the BeeWatch data is not preference data pur sang, we expect it to be a reasonable signal thereof. Testing this assumption, for example by conducting an investigation of the 7 plant species identified from BeeWatch data as the most widely used by bumblebees in UK gardens (constituting > 3% of plant species used; see Suppl. Appendix Table 2), using a standardised protocol involving timed observations of specific numbers of flowers^30–32^, would be productive. This could take the form of a new citizen science initiative, professional investigation, or possibly conducted using data from other studies, where such data is available.

Earlier work evaluating the BeeWatch citizen science scheme^33^ drew out the importance of identification tools offered as a starting point for wider learning and exploration of one’s own gardening or nature space^18,34^. We suggest that simple (pollinator) labelling can be used as an entry point for this. Yet, creatively acknowledging levels of pollinator friendliness and complexities, by using intuitive visual interfaces for exploring pollinator-plant interactions or deploying recommender systems for suggesting collections of plants that will support a group of pollinators through the season, as pioneered by the Planting for Pollinators scheme^33,34^, would open the opportunity for individual transformative learning journeys^35^. It is these journeys that connect people to pollinating insects and, by extension, to their plight. Furthermore, we know that this process can lead to remarkable changes in garden management and initiation of positive environmental actions by individuals^33^.

We advocate the use of dynamic systems using filter-based data presentation approaches, which make use of up-to-date information to foster positive ecological thinking and action. Moreover, by using citizen science data captured at the national level to populate such systems, this would provide planting recommendations that reflect current knowledge and the complexities of pollinator friendliness, and ultimately help pollinating insect populations around the globe.

## Methods

### Pollinator- and bumblebee-friendly practitioner plant data

Practitioner plant suggestions were compiled from 23 widely available UK sources (Suppl. Appendix Table 1 —sources that were readily available to the general public through internet and library searches for “pollinator-friendly plants” and “bumblebee-friendly plants”) that detailed plants identified as being good for pollinators in general or bumblebees in particular. To evaluate the usefulness of pollinator- and bumblebee-friendly practitioner plant lists we ranked each plant based on the number of literature sources where it occurred as a recommended species. In some instances, plants have been broken down to the species level, whereas in other cases only genera have been used, reflecting the degree of specificity we found in practitioner recommendations. For similar reasons, we did not include all the cultivars available for certain plant species that have undergone horticultural breeding and selection. This enabled us to produce two ranked plant lists derived from literature sources: (i) plants which were identified as pollinator-friendly (9 sources; see Suppl. Appendix Table 1 [sources numbered 1–9]); and (ii) plants which were identified as being specifically good for bumblebees (14 sources, see Suppl. Appendix Table 1 [in bold font, sources numbered 10–23]). Some plants classed as being good for bumblebees may have been missed from our combined list if they were recorded in academic journal sources, which we did not access as we concentrated on sources that were widely available to the public. However, we believe this number of plant species to be small, particularly as we found many of the same plants mentioned with increasing frequency the more plant lists we consulted.

### BeeWatch—an online bumblebee recording citizen science scheme

BeeWatch is an online citizen science initiative developed by the University of Aberdeen and the Bumblebee Conservation Trust in 2011, and has been co-ordinated by the University of Aberdeen. Members of the public submit photographs of bumblebees with details on location, date of sighting and plant species used (if applicable) via the online interface. Further details about BeeWatch can be found in Van der Wal et al. 2016^30^. When specifying which plant species was being utilised by a bumblebee, recorders had the option of free data entry or they could choose a plant from the compiled list generated from 23 different sources (see Suppl. Appendix Table 1). Since the emphasis of BeeWatch has been on learning, exploration and positive action, its biological records are opportunistic rather than gathered through structured recording processes that take into account factors such as recording effort (timed searches^36^), plant-species specific sampling, prevailing weather conditions and quantification of the floral resources (to determine whether flower abundance could affect plant use). However, BeeWatch data do represent a ‘snapshot’ of plant use by bumblebees across UK gardens and greenspaces as observed by participants at any one time over a 6-year period.

### Range and diversity of plants used by bumblebees

Data was downloaded from the BeeWatch system at the beginning of July 2017 and covered the period from the initiation of the ‘live’ BeeWatch program on 25 August 2011 until 30 June 2017. For the purposes of these analyses, we separated our data into two groupings: the 16 true bumblebee species (see Table 1 for details) and the six cuckoo bumblebee species (see Suppl. Appendix Table 3 for details). We separated true bumblebee species and cuckoo bees, because cuckoos are known to employ a different life strategy to that of true bumblebees and hence use forage plants in a different way^37^. This generated two lists of plant species (see below for details) used by (i) all true bumblebees and (ii) all cuckoo bumblebees.

We collated all BeeWatch data where a plant species that had been used by a bumblebee was recorded. We ranked each plant by the number of times it was mentioned in the BeeWatch database, then counted the number of times a specific plant was mentioned as being use by a specific bumblebee. By then dividing these numbers by the total number of records for each bumblebee species we were able to produce proportion data on plant use by individual bumblebee species. We also utilized the overall numbers of plants used to derive the proportional use of individual plants by all bumblebee species combined.

### Statistical analyses

Pearson’s correlations were performed to examine relationships between: (i) the number of sources—for each of the plants mentioned in one or more of the 23 available practitioner recommendation sources—which identified a plant as pollinator-friendly (9 sources) with the number of sources recommending it (i.e. recommendation frequency) as bumblebee-friendly (14 sources); (ii) the recommendation frequency of plants as pollinator-friendly (9 sources) with the frequency of plant occurrence in the BeeWatch database, and; (iii) the recommendation frequency of plants as bumblebee-friendly (14 sources) with the frequency of plant occurrence recorded in the BeeWatch database. This enabled us to determine how similar the lists of all pollinator-recommended plants and those specifically recommended as bumblebee-friendly were, whilst also comparing practitioners’ recommendations with actual patterns of plant use recorded by BeeWatch participants.

We estimated the mean number of different plant species that could be expected to be used by the different bumblebee species by using rarefaction analysis, as this procedure accounts for the varying sample sizes apparent in the BeeWatch data. We randomly sub-sampled 10 bumblebee plant choices, without replacement, from our BeeWatch plant use database, using 100 repeats of the procedure. Using a sample size of 10 meant that we were unable to make predictions for bumblebee species in which we had less than ten records, i.e. the brown-banded carder and the red-shanked carder bees and the ruderal bumblebee. Pearson’s correlations were used to determine if there was a correlation between the number of months in which different bumblebee species were active and the mean number of different plant species they would be expected to use, as determined by the rarefaction analysis.

All analyses were carried out in program R version 3.4.0^38^, using the vegan package version 2.4–3^39^ for rarefactions. The map in Fig. 1a contains data from BeeWatch (2011–2017) and OS data Crown copyright and database right (2019) and was generated by H. Anderson using ArcGIS Desktop 10.7 Esri Inc. 1999–2018.

## Supplementary information

Supplementary Tables.
