# Supplementary material for: Citizen science data reveals the need for keeping garden plant recommendations up-to-date to help pollinators
Source: Sci Rep. 2020 Nov 24;10:20483. doi: 10.1038/s41598-020-77537-6 (PMC7686498; doi:10.1038/s41598-020-77537-6)
Supplement: Supplementary file 1 — Supplementary Tables. [file 41598_2020_77537_MOESM1_ESM.docx]

**Citizen science data reveals the need for keeping garden plant recommendations up-to-date to help pollinators**

*Helen B Anderson^1^, Annie Robinson^1^, Advaith Siddharthan^2^, Nirwan Sharma^2^, Helen Bostock^3^, Andrew Salisbury^3^, Stuart Roberts^4^, René van der Wal^1,5^

^1^ School of Biological Sciences, University of Aberdeen, 23 St Machar Drive, Aberdeen, UK

^2^ Knowledge Media Institute, The Open University, Milton Keynes, UK

^3^ RHS Garden Wisley, Nr Woking, United Kingdom, GU23 6QB

^4^ CAER, School of Agriculture, Policy and Development, University of Reading, Reading, UK

^5^ Swedish University of Agricultural Sciences (SLU), Department of Ecology, Ulls väg 16, 75651 Uppsala, Sweden

*Corresponding author; email: [helen.anderson@abdn.ac.uk](mailto:helen.anderson@abdn.ac.uk)

**Appendix Table 1** Data sources listing plant species that are good for pollinators in general (normal font, source numbers 1-9) or for bumblebees in particular (bold font, source numbers 10-23). These sources were used to compile a plant list of 465 species, which were used to compare with plants used by bumblebees in records submitted to BeeWatch.

| **Source number** | **Name** | **URL or publishing source** |
| --- | --- | --- |
| 1 | RHS Garden Plants | https://www.rhs.org.uk/science/pdf/conservation-and-biodiversity/wildlife/rhs_perfectforpollinators_plantlist-jan15.pdf |
| 2 | RHS Wild Flowers | https://www.rhs.org.uk/science/pdf/conservation-and-biodiversity/wildlife/rhs_perfectforpollinators_wildflowerlist-jan15.pdf |
| 3 | Get Bristol Buzzing | http://www.avonwildlifetrust.org.uk/getbristolbuzzing |
| 4 | Defra - Bees Needs | http://www.wildlifetrusts.org/bees-needs |
| 5 | Plants and Planting Plans for a Bee Garden | Little M 2012. Plants and planting plans for a bee garden. Spring Hill, Oxford, UK. |
| 6 | Natural Garden | Foley C 2009. The Natural Garden Handbook. New Holland Publishers (UK) Ltd, London, UK. |
| 7 | Wildlife Gardener | Bradbury K 2013. The Wildlife Gardener creating a haven for birds, bees and butterflies. Kyle Books, London, UK. |
| 8 | RHS and Wildlife Trusts | Tait M (ed.) 2006. Wildlife Gardening for Everyone. Think Publishing Limited, UK. |
| 9 | Hymettus – BWARS joint initiative | http://www.hymettus.org.uk/downloads/Info_sheets_2010/Garden%20buzz6[1].pdf  http://www.hymettus.org.uk/downloads/Info_sheets_2010/14_Gardening_Spring_1col_infosheet.pdf  http://www.hymettus.org.uk/downloads/Info_sheets_2010/15_Gardening_Summer_1col_infosheet.pdf  http://www.hymettus.org.uk/downloads/Info_sheets_2010/16_Gardening_Autumn_1col_infosheet.pdf  http://www.bwars.com/sites/www.bwars.com/files/info_sheets/Winter_active-bumblebees_infosheet.pdf  http://www.bwars.com/sites/www.bwars.com/files/info_sheets/03_Bombus_hypnorum_20120321.pdf  http://www.bwars.com/sites/www.bwars.com/files/info_sheets/18_Bombus_distinguendus_20100922.pdf |
| **10** | **Bumblebees** | **Free J and Butler C 1959. Bumblebees. New Naturalist, Collins, London, UK.** |
| **11** | **Bumblebees** | **Benton T 2009. Bumblebees. New Naturalist, Harper Collins Publishing Ltd, Glasgow, UK.** |
| **12** | **Warwickshire Bumblebees by Steven Falk, 2011** | **http://www.stevenfalk.co.uk/files/21577/warwickshiresbumblebees.pdf** |
| **13** | **Bumblebee.org** | **http://www.bumblebee.org/flowerlist.htm** |
| **14** | **Plants for Bees** | **Kirk WDJ & Howes FN 2012. Plants for Bees. A guide to the plants that benefit the bees of the British Isles. International Bee Research Association, UK.** |
| **15** | **Bumblebee Conservation Trust - BeeKind** | **http://beekind.bumblebeeconservation.org/finder** |
| **16** | **The Wildlife Trusts - Garden Bumblebees** | **http://www.wildlifetrusts.org/sites/wt-main.live.drupal.precedenthost.co.uk/files/files/Wildlife%20Gardening/GardenBumblebees.pdf** |
| **17** | **Bumblebee Conservation Trust – Managing Your Land** | **https://bumblebeeconservation.org/get-involved/managing-your-land/** |
| **18** | **Dave Goulson** | **http://www.sussex.ac.uk/lifesci/goulsonlab/resources/flowers**  **Goulson D 2010. Bumblebees; their behaviour, ecology and conservation. Oxford University Press, Oxford, UK.** |
| **19** | **Friends of the Earth** | **https://www.foe.co.uk/bees** |
| **20** | **DEFRA/The Wildlife Trusts** | **http://www.wildlifetrusts.org/sites/default/files/6192_defra_info_sheet_gardens_final.pdf** |
| **21** | **BBCT blog: Bumblebees and flowers a complex relationship** | **https://bumblebeeconservation.org/news/anthonys-blog/bumblebees-and-flowers-a-complex-relationship** |
| **22** | **Edwards Field Guide to Bumblebees** | **Edwards M and Jenner M 2009. Field Guide to the Bumblebees of Great Britain and Ireland (revised edition). Ocelli, UK.** |
| **23** | **Buglife Garden flowers for bumblebees** | **https://www.buglife.org.uk/sites/default/files/Plants%20for%20bees.pdf** |

**Appendix Table 2** The top 25 plant species, ranked by their frequency of occurrence, from the data sources detailed in Table 1 and categorised as pollinator-friendly or only bumblebee-friendly and the top plants used by all 16 true bumblebee species, as recorded by BeeWatch participants between August 2011 and June 2017. For pollinator- and bumblebee-friendly plants the frequency of occurrence refers to the number of sources where a plant was recommended (data obtained from the 23 literature sources detailed in Appendix Table 1). For bumblebee-friendly plants the frequency of occurrence refers to the number of sources where a plant was recommended (data obtained from the 14 literature sources in Appendix Table 1 that were identified as being specifically good for bumblebees (sources 10-23). For BeeWatch plants the proportion is the number of times that plant species was recorded divided by the total number of plant species in the BeeWatch database. Bold font indicates plant species that appear in both the bumblebee-friendly plant list recommended by practitioners and in the BeeWatch database.

| **Rank** | **Pollinator-friendly plants** | **Frequency of occurrence** | **Bumblebee-friendly plants** | **Frequency of occurrence** | **BeeWatch** | **Proportion** |
| --- | --- | --- | --- | --- | --- | --- |
| 1 | *Centaurea* spp. | 8 | *Salix* spp. | 12 | ***Lavandula* spp.** | **0.08** |
| 2 | *Digitalis* spp. | 8 | ***Digitalis* spp.** | **11** | *Geranium* spp. | 0.04 |
| 3 | *Geranium* spp. | 8 | ***Lavandula* spp.** | **11** | *Cirsium* spp. | 0.04 |
| 4 | *Lamium* spp. | 8 | ***Symphytum officinale*** | **11** | ***Cotoneaster* spp.** | **0.03** |
| 5 | *Lonicera* spp. | 8 | ***Allium schoenoprasum*** | **10** | ***Allium schoenoprasum*** | **0.03** |
| 6 | *Rubus fruticosus* agg. | 8 | ***Calluna vulgaris*** | **10** | ***Centaurea* spp.** | **0.03** |
| 7 | *Sedum spectabile* | 8 | ***Centaurea* spp.** | **10** | *Rubus fruticosus* agg. | 0.03 |
| 8 | *Thymus* spp. | 8 | *Lamium* spp. | 10 | *Allium* spp. | 0.02 |
| 9 | *Antirrhinum majus* | 7 | *Malus domestica* | 10 | *Buddleja* spp. | 0.02 |
| 10 | *Borago officinalis* | 7 | *Rubus idaeus* | 10 | ***Digitalis* spp.** | **0.02** |
| 11 | *Campanula* spp. | 7 | ***Trifolium repens*** | **10** | *Trifolium* spp. | 0.02 |
| 12 | *Centaurea cyanus* | 7 | *Echium vulgare* | 9 | ***Calluna vulgaris*** | **0.02** |
| 13 | *Crocus* spp. | 7 | *Nepeta* spp. | 9 | ***Symphytum officinale*** | **0.02** |
| 14 | *Hedera* spp. | 7 | *Rosmarinus officinalis* | 9 | *Cirsium palustre* | 0.01 |
| 15 | *Lavandula* spp. | 7 | ***Thymus* spp.** | **9** | *Salvia* spp. | 0.01 |
| 16 | *Mahonia* spp. | 7 | ***Trifolium pratense*** | **9** | ***Centaurea cyanus*** | **0.01** |
| 17 | *Origanum* spp. | 7 | *Aster* spp. and hybrids | 9 | *Hebe* spp. | 0.01 |
| 18 | *Ribes* spp. | 7 | ***Cotoneaste*r spp.** | **8** | *Scabosia* spp. | 0.01 |
| 19 | *Salix* spp. | 7 | *Crocus* spp. | 8 | *Sedum spectabile* | 0.01 |
| 20 | *Scabosia* spp. | 7 | *Dipsacus fullonum* | 8 | ***Origanum* spp.** | **0.01** |
| 21 | *Stachys* spp. | 7 | *Linaria* spp. | 8 | *Rhododendron* spp. | 0.01 |
| 22 | *Ajuga reptans* | 6 | *Lonicera* spp. | 8 | ***Trifolium repens*** | **0.01** |
| 23 | *Allium* spp. | 6 | *Lotus* spp. | 8 | ***Thymus* spp.** | **0.01** |
| 24 | *Aster* spp. and hybrids | 6 | ***Origanum* spp.** | **8** | ***Trifolium pratense*** | **0.01** |
| 25 | *Cirsium* spp. | 6 | *Pulmonaria* spp. | 8 | *Papaver* spp. | 0.01 |

**Appendix Table 3** Rank order of the top 25 plant species used by all cuckoo bumblebee species based on data from the citizen science programme BeeWatch. For each plant, its relative abundance (i.e. proportion) on the BeeWatch database was calculated, for all bumblebee species combined (‘All’) and for each of the 6 cuckoo bumblebee species. Coloured bars are included to aid interpretation, whereby the top plant of a certain bumblebee species attracted the widest colour bar, and the colour bars of the other plants are proportional to that top plant. Where the bar is not the full width of the column, a species not in the overall top 25 list was the most widely used for that bumblebee species. The total number of plants observed (for all cuckoo bumblebees combined and individual species respectively) is provided in the second row from the bottom. The bottom row details the proportion of plants (from a total of 110) used each individual cuckoo bumblebee species. Data compiled from information recorded by BeeWatch participants between August 2011 and June 2017 (956 cuckoo bumblebee-plant interactions).
